# Reduced occurrence of alpha waves during resting state predicts high attention‐deficit/hyperactivity disorder traits in young adults

**DOI:** 10.1002/jcv2.70083

**Published:** 2026-02-21

**Authors:** Julio Rodriguez‐Larios, Ümit Aydin, Gráinne McLoughlin

**Affiliations:** ^1^ Brunel University of London London UK; ^2^ School of Psychology & Clinical Language Sciences University of Reading Reading UK; ^3^ Social Genetic & Developmental Psychiatry Centre Institute of Psychiatry Psychology & Neuroscience King's College London London UK

**Keywords:** ADHD, alpha oscillations, biomarkers, EEG, twin study

## Abstract

**Background:**

Attention‐deficit/hyperactivity disorder (ADHD) is a neurodevelopmental condition with significant cognitive and social impacts. Identifying reliable biomarkers for ADHD is crucial for developing personalised therapies. Electroencephalography (EEG) alpha oscillations (8–12 Hz) have been suggested as a potential biomarker, but findings have been inconsistent.

**Methods:**

This study aimed to investigate whether alpha oscillations in young adulthood are associated with high ADHD traits using EEG data from a large twin sample (*N* = 556) enriched with participants with ADHD and autism. We assessed whether alpha oscillations during rest were associated with high ADHD traits. In addition, we used twin modelling to estimate the heritability of EEG alpha measures and their relationship with ADHD traits.

**Results:**

Results showed that relative alpha peak amplitude was a significant predictor of ADHD traits when controlling for other factors such as age, sex and autistic traits. Specifically, we found that for each unit decrease in relative alpha peak amplitude (*z*‐scored), the likelihood of being in the high ADHD trait group increased by approximately 26%. Further analysis suggested that group differences were due to a reduced occurrence (but not amplitude) of oscillatory bursts in the alpha range. Finally, our twin modelling results suggested that although these alpha measures are heritable, the genetic factors contributing to individual differences in alpha measures and ADHD traits were largely independent.

**Conclusion:**

Together, these findings suggest that reduced alpha oscillations, particularly the occurrence of alpha bursts, may serve as a potential biomarker for ADHD. Our results may have implications for neuromodulation therapies targeting alpha rhythms in ADHD, such as neurofeedback and transcranial alternating current stimulation.

## Key Points

**What's known:**

- ADHD has been linked to atypical brain activity, with Electroencephalography (EEG) alpha oscillations (8–12 Hz) suggested as a potential biomarker. However, most of previous studies have relied on small samples and yielded inconsistent results.

**What's new:**

- This study demonstrates that young adults with high ADHD traits show a significant reduction in the occurrence (but not the amplitude) of alpha waves independent of autistic traits.

**What's relevant:**

- Findings support the use of EEG alpha dynamics—especially wave occurrence—as a potential biomarker for ADHD. This has important implications for the development of personalised neuromodulation therapies targeting alpha rhythms such as neurofeedback or transcranial alternating current stimulation.

## INTRODUCTION

Attention‐deficit/hyperactivity disorder (ADHD) is a neurodevelopmental condition characterised by pervasive patterns of inattention, hyperactivity, and impulsivity that is estimated to affect approximately 5% of adults worldwide (McCarthy et al., 2012; Polanczyk et al., 2007). ADHD has a significant impact on individuals' quality of life, mental health and social relationships (Adamou et al., 2013; Aduen et al., 2018; Aydin et al., 2022a; Capp et al., 2023). While psychopharmacological treatments, particularly stimulant medications, are considered the most effective interventions for managing ADHD symptoms (Cortese et al., 2018; Crescenzo et al., 2017; Skirrow et al., 2014), their use is frequently accompanied by side effects such as loss of appetite, sleep problems, and mood disturbances (Cascade et al., 2010). Although non‐pharmacological treatments show high promise, the evidence of their success is still limited (Nimmo‐Smith et al., 2020).

Recent advancements in precision psychiatry and neuromodulation have paved the way for developing personalised therapies for ADHD (Arns, 2012; Voetterl et al., 2023). In this regard, neuromodulation techniques, such as neurofeedback and non‐invasive brain stimulation, seem promising alternative or complementary treatments (Arns et al., 2014; Boetzel & Herrmann, 2021; Frohlich & Riddle, 2021). These approaches aim to directly modulate brain activity to improve symptomatology by promoting neuroplastic changes (Arns et al., 2014; Veniero et al., 2015; Vossen et al., 2015). In this context, EEG signatures can act as different classes of biomarkers that are relevant for neuromodulation (FDA‐NIH Biomarker Working Group, 2016; Michelini et al., 2022). For instance, EEG metrics could serve as a monitoring and/or response biomarkers that would allow to track within‐person changes after neuromodulation that co‐vary with symptoms (Klooster et al., 2024; Riddle & Frohlich, 2023).

The study of brain oscillations in ADHD has emerged as a particularly promising approach for identifying biomarkers relevant for neuromodulation (Günther et al., 2022; McLoughlin, Makeig, et al., 2014; Riddle & Frohlich, 2021). Neural oscillations, as measured by electroencephalography (EEG), reflect the coordinated firing of neuronal populations and have been implicated in various cognitive and behavioural processes (Klimesch, 1999b; von Stein & Sarnthein, 2000). The most widely studied oscillatory measure in ADHD is the theta/beta ratio, with previous literature suggesting a higher ratio between theta (4–7 Hz) and beta (13–30 Hz) power in ADHD (Arns et al., 2013; McLoughlin et al., 2022). However, recent evidence has challenged the reliability and specificity of this finding (for a review, see McLoughlin et al., 2022). As an alternative, alpha oscillations (8–12 Hz) have emerged as an EEG characteristic of interest in ADHD research, with several studies reporting significant reductions in individuals with ADHD (Cañigueral et al., 2022; Deiber et al., 2020; Loo et al., 2009; Voetterl et al., 2023; Woltering et al., 2012).

Alpha oscillations fulfil several conditions to become a relevant ADHD biomarker. First, alpha oscillations have the highest test‐retest reliability (Gudmundsson et al., 2007; Mathewson et al., 2015; Näpflin et al., 2007) and signal‐to‐noise ratio (Klimesch, 1999a, 2012) when compared to other brain rhythms. Secondly, alpha oscillations could offer a mechanistic explanation for cognitive deficits in ADHD (Pievsky & McGrath, 2018), given their essential role in inhibitory control and arousal regulation (Bazanova & Vernon, 2014; Codispoti et al., 2023; Klimesch et al., 2007). Lastly, alpha oscillations can be modified by neuromodulation techniques such as transcranial alternating current stimulation (tACS) (Vossen et al., 2015), transcranial magnetic stimulation (TMS) (Gregorio et al., 2022) and neurofeedback (Deiber et al., 2020), thereby emphasising its potential for treatment development (Holland, 2016).

In the present study, we investigated whether alpha oscillations are associated with high ADHD traits in a large EEG sample of twins (*N* = 556). Specifically, we compared relative alpha peak amplitude between individuals with high (*N* = 193) and low ADHD traits (*N* = 343) using a logistic regression analysis that controlled for covariates including age, sex and autistic traits. In addition, we assess whether putative differences in alpha peak amplitude are actually due to oscillatory activity (Donoghue et al., 2021) using a recently developed algorithm that detects oscillatory bursts in EEG (Rodriguez‐Larios et al., 2024; Rodriguez‐Larios & Haegens, 2023). Finally, we used twin modelling to assess the genetic and environmental contributions to variance in alpha oscillatory dynamics, allowing us to estimate the heritability of the relevant alpha measures and their relationship with ADHD traits.

## METHODS

### Participants

Ethical approval for the study was received from King's College London Psychiatry, Nursing and Midwifery Research Ethics Subcommittee (RESCMR‐16/17–2673). Data were collected as part of the Individual Differences in EEG in young Adults Study (IDEAS), which is a subsample of the Twins Early Development Study (TEDS) (Rimfeld et al., 2019). The sample consisted of 556 participants (267 males) with an average age of 22.43 (SD = 0.96 years) (119 Monozygotic (MZ) and 164 Dizygotic (DZ) pairs) (see full sample description at (Aydin et al., 2022a)).

### Questionnaires and trait characterisation

Participants were classified into high and low ADHD trait groups using both the Diagnostic Interview for ADHD in Adults (DIVA 2.0) and the Barkley Adult ADHD Rating Scale (BAARS‐IV). The BAARS provided a dimensional self‐report measure of symptom severity across daily contexts, whereas the DIVA is a structured clinical interview to assess onset, chronicity, and functional impairment according to DSM‐5 diagnostic criteria. Participants were classified as having high ADHD traits if they met DSM‐5 diagnostic criteria for adult ADHD based on DIVA 2.0 or if their BAARS‐IV total ADHD score was 39 or higher, reflecting mild to significant ADHD traits and related difficulties (Capp et al., 2023). This approach was followed to increase our sensitivity to detect ADHD traits. This resulted in 205 participants in the high ADHD traits group and 351 in the low ADHD traits group.

A similar approach was followed to control for autistic traits. In this way, the Autism Diagnostic Observation schedule 2 (ADOS‐2) and Social Responsiveness Scale‐2 (SRS‐2) were used to classify participants into those with high (*N* = 127) and low (*N* = 429) autistic traits. Individuals with high autistic traits were the ones that scored four or higher on the ADOS‐2 CSS (indicating behaviours consistent with an autism diagnosis) or had an SRS‐2 raw score of 68 or above (indicating mild to severe difficulties with social behaviour related to autism). Those who did not meet these criteria were classified as having low autistic traits (Capp et al., 2023).

### EEG recording and analysis

EEG was recorded with a mobile wireless 64‐channel (10‐10 montage) system (Cognionics, San Diego, CA; Ag/AgCl electrodes, with a sampling rate of 500 Hz). The dataset contained both resting state (eyes open and eyes closed) and task EEG data (Aydin, Gyurkovics, et al., 2023). Three minutes long eyes closed resting state EEG data was used in this study. This choice was based on previous literature showing higher reproducibility of eyes closed resting state EEG (Duan et al., 2021).

EEG analysis was performed in MATLAB R2023a using custom scripts and EEGLAB (Delorme & Makeig, 2004). Data cleaning was performed using an automatic pre‐processing pipeline based on EEGLAB functions. First, data were resampled to 250 Hz, re‐referenced to common average and filtered between 1 and 30 Hz. Abrupt noise in the data was removed using the Artefact Subspace Reconstruction method with a cut‐off value of 20 SD (Chang et al., 2019). Noisy channels were detected automatically with the EEGLAB function *clean_channels* using a threshold of 0.6 and were later interpolated. The average number of interpolated channels was 9.5 (SD = 3.6) and no subject was excluded based on the number of interpolated channels. Independent component analysis (ICA) and an automatic component rejection algorithm (Pion‐Tonachini et al., 2019) were used to discard components associated with muscle activity, eye movements, heart activity or channel noise (threshold = 0.8). The average number of rejected components was 2.5 (SD = 2.3). Continuous data was divided into 2 s epochs (50% overlap) and epochs that exceeded an amplitude of ±100 mV were removed (average number of rejected epochs = 18). Subjects with more than 50% of rejected epochs were excluded from subsequent analysis (*N* = 20).

For the estimation of relative alpha peak amplitude and the theta/beta ratio, the spectrum between 1 and 30 Hz (in steps of 0.5 Hz) was estimated using a short‐time Fourier transform as implemented through the MATLAB function *spectrogram* (sliding window of 2 s with 50% overlap). Individual alpha peak was estimated as the local maximum between 7 and 14 Hz using the *findpeaks* MATLAB function. Subjects that did not show an alpha peak in a specific electrode were excluded from the statistical analysis. In this regard, 23 subjects did not show an alpha peak in at least one electrode. Relative alpha peak amplitude was defined as the amplitude of the individual alpha peak of the normalised spectrum (i.e., z‐scored over the frequency dimension). The theta/beta ratio was estimated from the Cz electrode as the average power in the 4–7 Hz range (theta) divided by the power in the 13–30 Hz range (beta) (Arns et al., 2013).

In order to assess whether putative differences in relative alpha peak amplitude were due to oscillatory activity, a recently developed algorithm was adapted (Rodriguez‐Larios et al., 2024; Rodriguez‐Larios & Haegens, 2023). In short, EEG data is first transformed to the time‐frequency domain using Morlet wavelets (6 cycles width) as implemented in (Caplan et al., 2015). The frequency resolution was 1 Hz (between 1 and 30 Hz). Oscillatory bursts are defined as time points in which the amplitude at a specific frequency exceeds estimate of aperiodic activity (e.g., 1/f trend) for at least one full cycle. The 1/f trend is estimated by fitting a straight line to the spectrum in the log‐log space per electrode across all time points and epochs. Only oscillatory bursts that had their highest spectral peak in the alpha range (7–14 Hz) were selected. This algorithm allows for disentangling the quantity of time in which oscillatory activity is present (i.e., oscillatory burst coverage) and the amplitude of the burst (which is estimated as the oscillation maximum peak prominence in the time domain).

### Statistical analysis

A logistic regression model was employed to investigate the association between EEG alpha oscillations and the likelihood of having high ADHD traits. The analysis was conducted using a generalised linear mixed‐effects model (GLMM) with a logit link function in MATLAB 2023a (*fitglme* function). To control for potential confounding factors, the model included additional fixed effects for age, sex and autistic traits (binary: high vs. low). To account for clustering within the sample due to relatedness (i.e., twins), random intercepts were included in the model for each pair of twins (Aydin, Cañigueral, et al., 2023; Malone et al., 2014). Only the association between alpha metrics and ADHD traits will be discussed as the rest of the predictors were only included as controls. In line with previous literature (Chu et al., 2020), effect sizes were estimated through the odds ratio, which was calculated by exponentiating the logistic regression coefficients (*β*). In order to assess significance, t‐values for fixed effects were calculated as the ratio of the estimated coefficient to its standard error.

In order to test the relationship between ADHD symptomatology and EEG metrics, we performed a linear mixed model as implemented in MATLAB (fitlme function). For this purpose, we used the total score of the BAARS questionnaire as a dependent variable. Like in the logistic regression analysis, the model included additional fixed effects for age, sex and autistic traits (binary: high vs. low) and random intercepts for each pair of twins. Continuous variables (BAARS, alpha amplitude, age) were normalised (z‐scored) prior to analysis to facilitate interpretation of regression coefficients in terms of effect size (i.e., the change in the dependent variable in standard deviations associated with a one standard deviation change in the predictor).

Correlations were computed through Pearson correlation coefficient and multiple comparisons correction was implemented through the False Discovery Rate (Benjamini & Hochberg, 1995). Outliers were detected and rejected before all statistical analyses. Outliers were defined as elements more than three scaled median absolute deviations from the median (see *isoutlier* function in MATLAB 2023a).

### Twin modelling

We applied genetic multivariate liability threshold models based on the principles that monozygotic (MZ) twins share 100% of their genetic influences, while dizygotic (DZ) twins share 50%, and both groups share common environmental factors equally (Neale & Maes, 2004). These models treat high ADHD traits and high autistic traits as dichotomous variables and use the MZ:DZ ratio of cross‐twin within‐trait correlations to partition the variation in EEG traits into standardised additive genetic variance (a^2^), common environmental variance (c^2^), and unique environmental variance, including measurement error (e^2^). The phenotypic correlation (Rph) between traits is calculated as the zero‐order correlation, while the MZ:DZ ratio of cross‐twin cross‐trait correlations is used to partition the covariation between traits (e.g., ADHD and relative alpha peak amplitude) to report genetic (Ra) correlations. Since the sample had a higher proportion of participants meeting ADHD or ASD criteria compared to the general population, we applied corrections to the standard twin model, as described in (Aydin et al., 2022b). Twin modelling was conducted using structural equation modelling in OpenMx (Boker et al., 2011), with likelihood‐based asymmetric 95% confidence intervals (CIs) estimated for all parameters. To examine associations between ADHD or autistic traits and specific EEG metrics, we ran bivariate models for relative alpha peak amplitude, burst alpha amplitude, and burst alpha coverage.

## RESULTS

### Relative alpha peak amplitude predicts ADHD traits and symptoms

**FIGURE 1:**
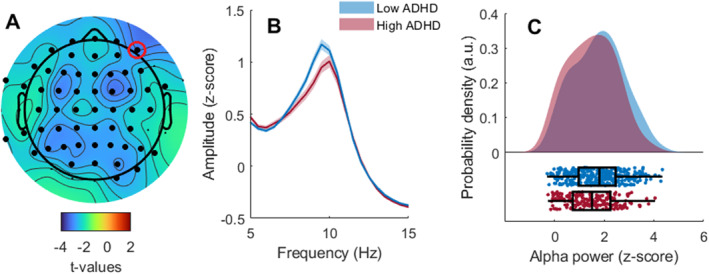
Association between relative alpha peak amplitude and ADHD traits. (A) Topographical plot depicting t‐values obtained when using relative alpha peak amplitude as predictor in the specified model. Black dots indicate significant electrodes (*p*‐value <0.05) after FDR correction and the red circle marks AF8, the most significant electrode (i.e., highest absolute *t*‐value). (B) Frequency spectrum (z‐scored) averaged across significant electrodes for low (blue) and high (red) ADHD trait groups. The shaded area depicts standard error. (C) Relative alpha amplitude for the low (blue) and high (red) ADHD trait groups. Each point depicts relative alpha amplitude for one subject across significant electrodes. Boxplots indicate the median and interquartile range per group. ADHD, attention‐deficit/hyperactivity disorder.

Logistic regression analysis revealed that relative alpha peak amplitude was a significant predictor of high ADHD traits (*p*‐values after FDR correction <0.05 in 55 out of 64 electrodes) (see Figure 1A). Specifically, participants with high ADHD traits showed a significant reduction in relative alpha peak amplitude when compared to the low ADHD trait group (see Figure 1B,C). The odds ratio in the electrode with the lowest *p*‐value (AF8) was 0.74, which means that for every unit of relative alpha amplitude (in z‐scores) the odds of being in the high ADHD trait group decreased by approximately 26% (t‐value (497) = −3.02; *p*‐value = 0.0026) (see Table 1). Subsequent analyses were performed in the electrode with the lowest *p*‐value (AF8; marked in Figure 1A).

**TABLE 1:** Logistic regression model for the prediction of ADHD traits (0 = low, 1 = high) from autistic traits (0 = low, 1 = high), age, sex and relative alpha peak amplitude at electrode AF8.

Predictor	*β*	SE	*t*‐value	*p*‐value	Odds ratio
Intercept	6.18	2.59	2.38	0.0172	487.23
Autistic traits	1.02	0.23	4.28	<0.001	2.78
Age	−0.29	0.11	−2.63	0.0087	0.74
Sex	0.03	0.21	0.17	0.8610	1.04
Relative alpha peak amplitude	−0.323	0.10	−3.02	0.0026	0.72

A dimensional analysis using the BAARS questionnaire (i.e., total ADHD score) showed that ADHD traits were negatively associated with relative alpha peak amplitude. Specifically, 1 SD increase in relative peak alpha amplitude in electrode AF8 was associated with a 0.09 SD decrease in the total ADHD score (*t*‐value (497) = −2.11; *p*‐value = 0.035) (see Table 2).

**TABLE 2:** Linear mixed model for the prediction of ADHD traits (BAARS total score) from autistic traits (0 = low, 1 = high), age, sex and relative alpha amplitude at electrode AF8.

Predictor	*Β*	SE	*t*‐value	*p*‐value
Intercept	0.18	0.05	3.41	0.0007
Autistic traits	−0.33	0.05	−6.19	<0.001
Age	−0.06	0.04	−1.37	0.1715
Sex	0.02	0.04	0.50	0.6158
Relative alpha peak amplitude	−0.09	0.04	−2.11	0.0355

In order to assess the specificity of our findings, we performed the same analyses with a commonly used EEG metric in the ADHD literature (i.e., theta/beta ratio; TBR). Unlike relative alpha amplitude, TBR was not a significant predictor of either high ADHD traits whether using the binary grouping derived from BAARS and DIVA (*t*‐value (497) = −0.52; *p*‐value = 0.60) or the continuous BAARS total score (*t*‐value (497) = −0.86; *p*‐value = 0.38).

### Assessing the putative oscillatory nature of alpha amplitude modulations

In order to assess whether the reported modulations of alpha amplitude in relation to ADHD traits were due to oscillatory activity, we employed a recently developed algorithm that estimates the occurrence and amplitude of oscillatory bursts (Rodriguez‐Larios et al., 2024; Rodriguez‐Larios & Haegens, 2023). For this analysis, we selected the frontal EEG electrode with the highest group difference as quantified by its t‐value (i.e., electrode AF8; see red circle in Figure 1).

**FIGURE 2:**
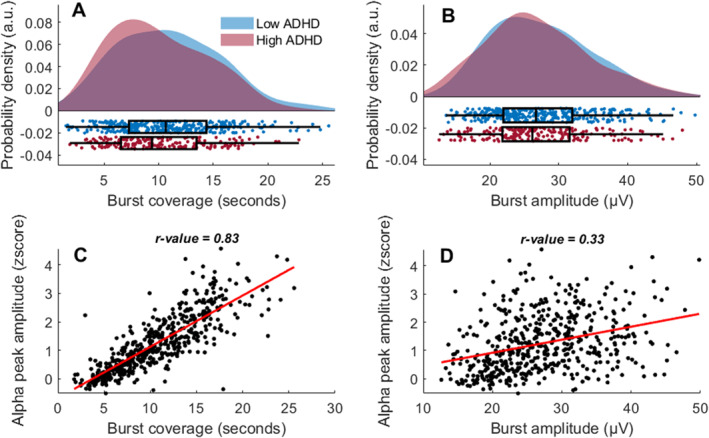
Alpha oscillatory burst differences between groups with high and low ADHD traits and their correlation with relative alpha amplitude. (A) Burst coverage for the low (blue) and high (red) ADHD trait groups. Each point depicts relative alpha amplitude for one subject for the selected frontal electrode. Boxplots indicate median and interquartile range per group. (B) Same as A but for burst amplitude. (C, D) Correlation between burst parameters (coverage and amplitude) and individual alpha peak amplitude (each point depicts data of one subject for the selected electrode). ADHD, attention‐deficit/hyperactivity disorder.

On the one hand, logistic regression analysis revealed a significant effect of oscillatory burst coverage (t‐value (499) = −2.28, *p*‐value = 0.0228) but not of oscillatory burst amplitude (*t*‐value (499) = 0.13, *p*‐value = 0.89) (see Table 3 for full model details and Figure 2A,B for depiction of results). The odds ratio for burst coverage was 0.94, which means that for every second of alpha oscillatory bursts in the resting state EEG, the odds of being in the high ADHD trait group decreased by approximately 6%.

**TABLE 3:** Logistic regression model for the prediction of ADHD trait (0 = low, 1 = high) from autistic trait (0 = low, 1 = high), age, sex, mean oscillatory burst alpha amplitude (i.e., amplitude) and mean oscillatory burst alpha coverage (coverage) at electrode AF8.

Predictor	*β*	SE	*t*‐value	*p*‐value	Odds ratio
Intercept	4.90	2.46	1.98	0.0472	134.93
Autistic traits	1.04	0.23	4.48	<0.001	2.84
Age	−0.23	0.10	−2.15	0.0316	0.79
Sex	0.04	0.20	0.22	0.8245	1.04
Amplitude	0.002	0.01	0.13	0.8901	1.00
Coverage	−0.06	0.02	−2.28	0.0228	0.94

On the other hand, a dimensional analysis (i.e., linear mixed model) showed that ADHD traits (as assessed with BAARS total score) were significantly associated with alpha burst coverage but not amplitude (see Table 4 for full model). Specifically, 1 SD increase in burst coverage was associated with a 0.10 SD decrease in BAARS score (*t*‐value (497) = −2.11; *p*‐value = 0.035) (see Table 4).

**TABLE 4:** Linear mixed model for the prediction of ADHD traits (BAARS total score) from autistic traits (0 = low, 1 = high), age, sex, mean oscillatory burst alpha amplitude (i.e., amplitude) and mean oscillatory burst alpha coverage (coverage) at electrode AF8.

Predictor	*β*	SE	*t*‐value	*p*‐value
Intercept	0.18	0.05	3.38	0.0007
Autistic traits	−0.33	0.05	−6.02	
Age	−0.06	0.04	−1.33	0.1821
Sex	0.002	0.04	0.05	0.9580
Amplitude	0.036	0.04	0.74	0.4553
Coverage	−0.10	0.04	−2.07	0.0387

Together, these results suggest that relative alpha amplitude modulations in relation to ADHD were mostly due to the occurrence of oscillatory bursts rather than their amplitude. In line with this idea, inter‐individual differences in relative alpha amplitude showed a higher correlation to burst coverage (*r*‐value = 0.83; *p*‐value <0.001; Figure 2C) than to burst amplitude (*r*‐value = 0.33; *p*‐value <0.001; Figure 2D).

### Genetic contributions of alpha parameters

We found statistically significant and high genetic contributions (a^2^) to the total variance for relative alpha peak amplitude and burst alpha coverage but not for burst alpha amplitude (Table 5). Common environment contributions (c^2^) were not significant. In terms of associations between ADHD traits and alpha measures, we observed significant negative phenotypic correlations (Rph) for relative alpha amplitude (−0.15) and burst alpha coverage (−0.12) but in both cases, the genetic correlations (Ra) were not significant (Table 5). Phenotypic and genetic correlations between autistic traits and the alpha measures were not statistically significant (see Table 6).

**TABLE 5:** Phenotypic (Rph) and genetic (Ra) correlations between ADHD trait and relative alpha amplitude, mean oscillatory burst alpha amplitude, and **mean oscillatory burst alpha coverage**.

	Rph	Ra	a^2^	c^2^	e^2^
Relative alpha peak amplitude	**−0.15** (−0.25, −0.05)	−0.14 (−0.31, 0.03)	**0.59** (0.48, 0.69)	0.00 (0.00, 0.28)	**0.41** (0.31, 0.52)
Burst alpha amplitude	−0.04 (−0.14, 0.05)	−0.05 (−1.00, 1.00)	0.38 (0.00, 0.61)	0.11 (0.00, 0.44)	**0.51** (0.39, 0.65)
Burst alpha coverage	**−0.12** (−0.22, −0.03)	−0.13 (−0.31, 0.05)	**0.59** (0.46, 0.69)	0.00 (0.00, 0.29)	**0.41** (0.31, 0.54)

*Note*: Standardised estimates of genetic (**a** ^**2**^), common (**c** ^**2**^) and nonshared (**e** ^**2**^) environment contributions to variance of the EEG measures. 95% confidence intervals are given in parentheses, and significant estimates are written in bold.

**TABLE 6:** Phenotypic (Rph) and genetic (Ra) correlations between autistic trait and relative alpha amplitude, mean oscillatory burst alpha amplitude, and **mean oscillatory burst alpha coverage**.

	Rph	Ra	a^2^	c^2^	e^2^
Relative alpha peak amplitude	−0.07 (−0.19, 0.05)	−0.09 (−0.30, 0.12)	**0.59** (0.26, 0.69)	0.00 (0.00, 0.28)	**0.41** (0.31, 0.52)
Burst alpha amplitude	−0.08 (−0.05, 0.20)	0.27 (−0.02, 1.00)	0.35 (0.00, 0.61)	0.13 (0.00, 0.44)	**0.51** (0.39, 0.65)
Burst alpha coverage	−0.11 (−0.23, 0.01)	−0.20 (−0.41, 0.02)	**0.59** (0.46, 0.69)	0.00 (0.00, 0.27)	**0.41** (0.31, 0.54)

*Note*: Standardised estimates of genetic (**a** ^**2**^), common (**c** ^**2**^) and nonshared (**e** ^**2**^) environment contributions to variance of the EEG measures. 95% confidence intervals are given in parentheses, and significant estimates are written in bold.

Taken together, these findings suggest that while relative alpha peak amplitude and alpha burst coverage are highly heritable, their modest phenotypic associations with ADHD traits are not explained by common genetic influences.

## DISCUSSION

The aim of this study was to investigate whether adults with high ADHD traits show alterations in alpha oscillations during resting state. For this purpose, we analysed a large twin EEG data set (*N* = 556) enriched for ADHD and autistic traits (Aydin et al., 2022a; McLoughlin, Palmer, et al., 2014). Our results showed that participants with high ADHD traits have significantly reduced relative alpha peak amplitude at rest across EEG electrodes. Specifically, logistic regression analysis revealed that for every unit of relative alpha peak amplitude (in *z*‐scores), the odds of being in the high ADHD trait group decreased by approximately 26%. Further analysis suggested that group differences in relative alpha amplitude were due to changes in the occurrence of oscillatory bursts in the alpha range rather than their amplitude. Crucially, unlike alpha metrics, the theta/beta ratio was not a significant predictor of ADHD traits, which is in line with recent reports (Arns et al., 2013; McLoughlin et al., 2022). Finally, our twin modelling analysis showed that although the relevant alpha metrics for ADHD are highly heritable (i.e., relative peak amplitude and burst coverage), their associations with ADHD traits are not explained by common genetic influences.

Although previous studies have shown that relative alpha amplitude during resting state is significantly reduced in populations with high ADHD traits (Deiber et al., 2020; Loo et al., 2009; Ponomarev et al., 2014; Woltering et al., 2012), this finding was not completely consistent with the literature (Bresnahan et al., 2006; Koehler et al., 2009; Poil et al., 2014; van Dongen‐Boomsma et al., 2010; Voetterl et al., 2023). These inconsistencies could be due to small sample sizes and the lack of control variables in previous studies. Our large and well‐characterised sample allowed us to clarify these inconsistencies by demonstrating that reduced alpha amplitude remains significantly associated with ADHD even when controlling for autistic traits, age and sex. In addition, by employing a recently developed algorithm (Rodriguez‐Larios et al., 2024; Rodriguez‐Larios & Haegens, 2023) we further characterise the origin of the reported alpha differences and control for the possible influence of aperiodic activity. This analysis suggested that alpha amplitude differences between high and low ADHD groups were more influenced by the occurrence of alpha bursts than by their amplitude. Nonetheless, it is important to highlight that relative alpha peak amplitude was a better predictor of ADHD traits than alpha burst coverage (i.e., greater effect size as quantified through the odds ratio). This could be because relative alpha peak amplitude is also influenced by other EEG parameters such as aperiodic activity and/or band power at other frequency bands (Donoghue et al., 2021) that are also affected in ADHD (Chen et al., 2024; Dakwar‐Kawar et al., 2024). Although the odds ratio for burst coverage indicates a smaller effect size than that for relative alpha peak amplitude, its identification as a potential ADHD biomarker remains important from a translational perspective. For example, in the context of neurofeedback, the use of oscillatory burst metrics (such as coverage) might be more effective for ADHD than general alpha amplitude estimates (Deiber et al., 2020) due to their higher degree of specificity (Enriquez‐Geppert et al., 2017). In support of this idea, one previous study suggests that alpha neurofeedback protocols actually modulate alpha burst occurrence rather than alpha amplitude per se (Ossadtchi et al., 2017).

Our twin modelling results confirmed the high heritability of EEG alpha measures, with particularly strong genetic contributions to relative alpha amplitude and burst alpha coverage. This finding aligns with previous reports indicating the high heritability of alpha oscillations' characteristics (Malone et al., 2014; Smit et al., 2006; van Beijsterveldt & van Baal, 2002). Despite the observed phenotypic correlations between ADHD traits and alpha oscillations, we did not detect significant genetic correlations between these measures. This suggests that the genetic factors influencing alpha dynamics may be at least partly independent from those contributing to ADHD liability, although limited power cannot be excluded. Importantly, such independence would not diminish the relevance of alpha oscillations as a mechanistic target; on the contrary, it could indicate that modulating alpha activity might benefit individuals regardless of their underlying genetic risk profile.

The identification of alpha oscillations as a possible biomarker for ADHD has important implications for therapy. If reduced alpha oscillations are present in ADHD, neuromodulation protocols that enhance alpha oscillations (such as tACS and neurofeedback) (Soriano et al., 2024; Vossen et al., 2015) may improve ADHD symptoms. Recent evidence suggests that specific alpha oscillatory patterns may also help guide treatment selection, potentially improving clinical outcomes through personalised approaches (Voetterl et al., 2023). In this direction, it has been shown that a neurofeedback protocol that increased alpha amplitude improved ADHD symptoms and attentional performance (Deiber et al., 2020). Similarly, a recent alpha tACS study has shown that repeated sessions of alpha tACS can improve ADHD symptoms (although in this case changes in EEG were not investigated) (Farokhzadi et al., 2020). Together, although more evidence is needed, neuromodulation protocols aimed at enhancing alpha oscillations in ADHD show significant promise. An important question for future research is whether individuals with ADHD who exhibit reduced alpha amplitude at rest would derive greater benefits from these therapies.

Our study has several limitations. First, we did not systematically compare alpha metrics with a myriad of other metrics that can be obtained from EEG as this was beyond the scope of this study. Therefore, we cannot claim that metrics based on alpha oscillations are the best EEG predictor of ADHD traits. Secondly, the here proposed link between ADHD traits and resting state alpha wave activity is purely correlational. Future studies using neuromodulation techniques capable of modulating alpha wave activity (Neurofeedback, tACS, TMS) are needed to establish a causal relationship. Third, it is unclear how exactly resting state alpha waves are mechanistically related to ADHD symptomatology. Although alpha oscillations have been shown to play a key role in arousal regulation and attentional control (Bazanova & Vernon, 2014; Codispoti et al., 2023; Klimesch et al., 2007), we did not assess whether these processes are correlated with alpha metrics during rest. In fact, the observed differences in the high ADHD trait group could be due to several factors known to affect alpha waves, ranging from anatomical differences (Kumral et al., 2022; Valdés‐Hernández et al., 2010) to modulations in resting‐state cognition (e.g., mind wandering levels) (Compton et al., 2019; Rodriguez‐Larios & Alaerts, 2020; Rodriguez‐Larios et al., 2021). In order to answer these questions, future studies would highly benefit from a combination of resting state EEG, questionnaires and a comprehensive battery of cognitive tests in well‐characterised clinical samples. Lastly, the relationship between alpha metrics and ADHD traits was modest for both the categorical and dimensional analyses. Therefore, rather than serving as a standalone diagnostic biomarker, alpha activity may be more appropriately conceptualised as a monitoring and/or response biomarker relevant for neuromodulation. In this regard, EEG alpha metrics could be potentially useful for tracking treatment‐related changes in ADHD. Nonetheless, future longitudinal and interventional studies are needed to determine whether changes in alpha activity reliably parallel clinical improvement.

In conclusion, this study shows that adults with high ADHD traits have reduced relative alpha amplitude at rest, primarily due to a decrease in the occurrence of alpha oscillatory bursts. While our findings support the idea that alpha oscillations could be a potential biomarker for ADHD, its consistency across different populations and usefulness for therapy is still to be tested. Future research should investigate the underlying mechanisms contributing to these differences and determine if individuals with lower alpha amplitude benefit more from targeted neuromodulation therapies.

## AUTHOR CONTRIBUTIONS

**Julio Rodriguez‐Larios**: Conceptualization; formal analysis; methodology; investigation; software; visualization; writing—original Draft. **Ümit Aydin**: Data curation, formal analysis, methodology; software; writing—review and editing. **Gráinne McLoughlin**: Conceptualization, funding acquisition; project administration; resources; methodology; supervision; writing—review and editing.

## CONFLICT OF INTEREST STATEMENT

The authors declare no conflicts of interest.

## ETHICAL CONSIDERATIONS

Ethical approval for the study was received from King's College London Psychiatry, Nursing and Midwifery Research Ethics Subcommittee on 21/04/2016 (RESCMR‐16/17–2673). Informed consent was obtained from all participants.

## Data Availability

The data that support the findings of this study are available from the author G.M. upon reasonable request.
